# Abdominal disseminated lesions following surgery for a testicular mucinous cystic neoplasm initially diagnosed as mucinous cystadenoma: a case report and literature review

**DOI:** 10.3389/fonc.2026.1731890

**Published:** 2026-04-22

**Authors:** Junjie Yang, Weida Li, Jian Wang, Jianchang Li

**Affiliations:** Department of Urology, Affiliated Hospital of Guangdong Medical University, Zhanjiang, Guangdong, China

**Keywords:** abdominal metastasis, case report, ovarian-type tumor, primary testicular mucinous cystadenoma, testicular tumor

## Abstract

This report describes a rare testicular mucinous cystic neoplasm in a 78-year-old man who underwent scrotal exploration and right radical orchiectomy. Histopathology of the resected specimen was interpreted as an ovarian-type mucinous cystadenoma with focal epithelial proliferation. Six months after surgery, follow-up imaging demonstrated multiple abdominal and peritoneal lesions suspicious for disseminated disease. Because the abdominal lesions were diagnosed radiologically and were not histologically sampled, metastatic disease could not be pathologically confirmed. In addition, immunohistochemical analysis of the orchiectomy specimen was not performed, and a gastrointestinal primary tumor was not definitively excluded by endoscopic evaluation. The case highlights the diagnostic challenges of distinguishing benign, borderline, malignant, and metastatic mucinous tumors involving the testis, and it emphasizes the importance of postoperative surveillance, comprehensive clinicopathologic correlation, immunohistochemistry, and exclusion of an extratesticular primary site.

## 1 Introduction

Primary mucinous tumors of the testis and paratestis are exceptionally rare ovarian-type epithelial neoplasms. Published cases have been classified, by analogy with ovarian mucinous tumors, as benign mucinous cystadenoma, borderline mucinous tumor, or mucinous cystadenocarcinoma (1). However, because mucinous tumors metastatic to the testis or paratesticular tissues may mimic a primary lesion, the diagnosis requires careful clinicopathologic correlation and exclusion of a metastatic gastrointestinal or other extratesticular primary tumor (2). This paper retrospectively analyzes a case of postoperative abdominal metastasis following primary testicular mucinous cystadenoma, contributing to the understanding of its diagnosis and management, thus improving patient prognosis and quality of life.

## 2 Case report

The patient, a 78-year-old male, presented with a six-month history of bilateral scrotal enlargement, without a sensation of heaviness or pain upon palpation. He had no history of scrotal trauma, familial, or genetic predisposition. Physical examination revealed bilateral scrotal enlargement without erythema. A firm, irregular mass measuring approximately 10x8x5 cm was palpable above the left testis, while a smooth, cystic mass about 8x8x5 cm was noted on the right side. The transillumination test was negative, and there was mild tenderness upon palpation. The right testis was not palpable.

Serum tumor markers revealed elevated carcinoembryonic antigen (CEA) at 42.49 ng/ml (normal range 0-5 ng/ml), cancer antigen 125 (CA-125) at 44.81 U/ml (normal range 0-35 U/ml), and carbohydrate antigen 19-9 (CA19-9) at 87.00 U/ml (normal range 0-27 U/ml). Beta-human chorionic gonadotropin (β-HCG) and lactate dehydrogenase (LDH) were within normal limits, with no significant abnormalities in other laboratory tests.

**Figure 1:**
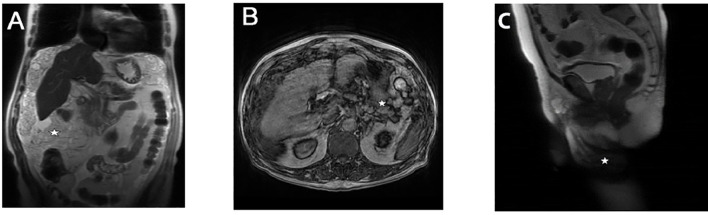
Preoperative MRI of the patient. **(A)** MRI shows multiple patchy low T1WI and high T2WI signals in the right scrotal area, with no high signal on DWI, and displaceent of both testes due to compression. **(B)** MRI indicates the presence of hydrocele in the left testis. **(C)** MRI reveals slightly enlarged lymph nodes in both inguinal regions, with no detectable tumors in the pelvic and abdominal cavities.

Ultrasonography indicated an enlarged left testis with a hypoechoic mass measuring approximately 4.1x2.3 cm, with clear boundaries and homogeneous internal echo. The right scrotum displayed a hypoechoic mass of about 8.2x6.4 cm with uneven internal echo and clear boundaries. No significant blood flow signals were observed in either testis, suggesting malignant lesions. MRI revealed multiple patchy low T1WI and high T2WI signals in both spermatic cords and the right scrotal area, with the largest lesion located on the right, measuring approximately 156x44x37 mm. Enhanced scans showed multiple septal and wall nodule enhancements. Both testes were displaced, with the left testis measuring about 30x16 mm and the right testis about 25x13 mm. Hydrocele signs were present on the left side, with slightly enlarged lymph nodes in both inguinal regions, but no tumors in the abdominal cavity (**Figure 1**).

The patient underwent a scrotal incision exploration and right radical orchiectomy. Intraoperatively, the left testicular tumor was found to be contiguous with the right scrotal tumor. The left testicular tumor was multilocular, containing pale yellow, jelly-like mucus, with no clear boundary with the testis. The right scrotal tumor was multilocular, containing brown, jelly-like mucus, and extended towards the right inguinal canal, terminating near the internal inguinal ring. The spermatic cord was severed at the internal ring, and the right testis along with the entire tumor was excised. The left testis and spermatic cord appeared normal with no apparent tumor presence.

**Figure 2:**
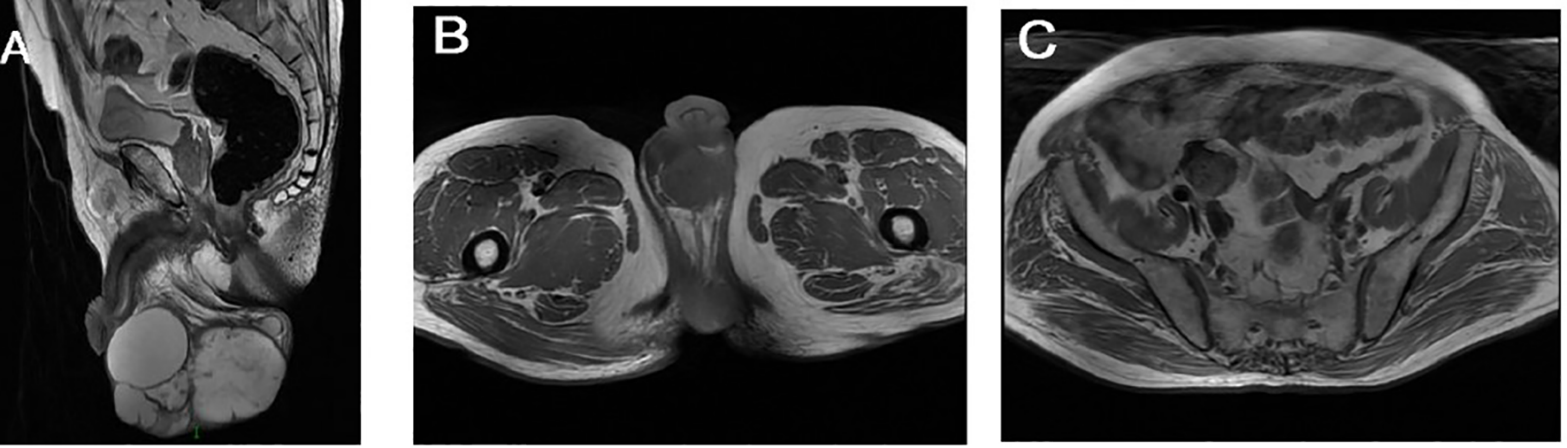
Pathology of the patient. **(A)** Gross specimen of the right testicular and epididymal tumor measuring 14.0×16.0×2.0 cm. **(B, C)** Microscopic images showing multicystic glands and fibrotic cyst walls lined with columnar mucinous epithelium.

Postoperative pathology revealed that the right testicular and epididymal tumor measured 14.0x16.0x2.0 cm in its gross specimen, with a multicystic, gray-brown, and gray-white appearance, containing translucent gelatinous material. Histological examination confirmed an ovarian epithelial-type tumor (mucinous cystadenoma with focal proliferation) (**Figure 2**). No immunohistochemical staining was performed on the available specimen, which limits distinction among benign, borderline, malignant, and metastatic mucinous lesions.

**Figure 3:**
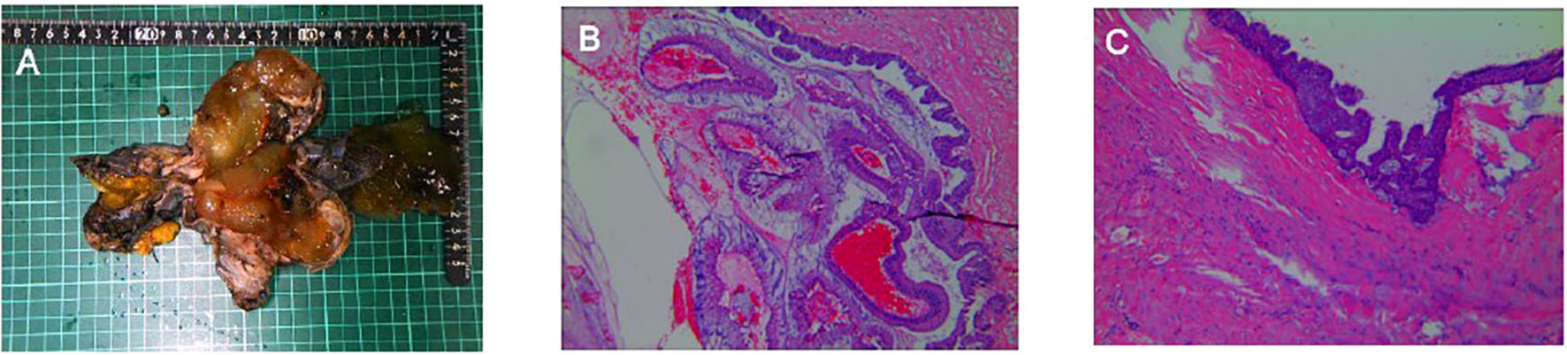
Six-month postoperative follow-up MRI of the patient. **(A, B)** MRI scans showing multiple lesions in the abdominal cavity and peritoneum. **(C)** The right testicular mass has been excised, demonstrating postoperative changes without any evidence of residual tumor or recurrence.

At the 6-month postoperative follow-up, MRI showed postoperative changes in the right scrotum without definite local recurrence but demonstrated multiple abdominal and peritoneal lesions suspicious for disseminated disease. These lesions were diagnosed radiologically only; no biopsy or surgical sampling was performed. Chemotherapy was recommended, but the patient declined further treatment. (**Figure 3**).

## 3 Discussion

According to the 2022 World Health Organization (WHO) classification of tumors of the urinary system and male genital organs, primary testicular mucinous cystadenoma is categorized under ovarian-type tumors of the collecting ducts and rete testis (1). The histogenesis of ovarian-type surface epithelial tumors in the testis and paratesticular tissue remains speculative. These tumors may arise from the mesothelium via a process of mucinous epithelial differentiation, or from mesothelial remnants or teratomatous mucinous epithelium. Some hypotheses suggest origins from Müllerian duct remnants, such as testicular appendages, or residual Müllerian duct remnants within the testicular tissue. Another theory posits that these tumors result from mesothelial metaplasia of the tunica vaginalis (analogous to female pelvic mesothelium). Thus, the possible origins include mucinous metaplasia of the mesothelium, Müllerian duct remnants in the appendix testis or other scrotal contents, and unilaterally differentiated teratomatous cells (2).

Most mucinous ovarian-type surface epithelial lesions described in the literature are benign or borderline, with only a few reported cases exhibiting invasive or malignant cytological features. To date, 31 cases of testicular or paratesticular ovarian-type mucinous tumors have been documented, including 9 mucinous cystadenomas, 15 borderline mucinous cystadenomas, and 7 mucinous cystadenocarcinomas (3). Notably, none of the 9 mucinous cystadenoma cases showed recurrence or metastasis. However, the occurrence of abdominal metastasis in the present case calls for a reassessment of our understanding of primary mucinous cystadenoma of the testis.

Thomas M et al. (4) reported nine cases of primary mucinous tumors of the testis and paratesticular tissue (8 cystic tumors containing gelatinous material and 1 paratesticular carcinoma with thickening of the tunica vaginalis), including one patient who died shortly after peritoneal spread. Alina Cornelia Iuga et al. (5) described a 71-year-old male with unilateral testicular cystadenocarcinoma with mucinous differentiation. The case displayed morphological characteristics similar to ovarian tumors, ranging from benign to malignant cytology, mucinous spillage, and areas of fibrosis, calcification, and inflammation. Immunohistochemical staining showed positive reactions for cytokeratin, carcinoembryonic antigen, epithelial membrane antigen, S-100, CA-125, CA 19-9, and cytokeratin CAM 5.2. These tumors exhibit behavior similar to ovarian tumors, typically smaller and more often unilocular with intestinal cell characteristics. Abdominal metastasis of testicular mucinous cystadenoma is exceedingly rare, generally presenting with multiple foci, prominent interstitial growth, and vascular invasion, with unclear mechanisms of occurrence (6). Research indicates that abdominal metastasis may be related to tumor aggressiveness and lymphatic spread. In this case, the patient’s abdominal metastasis likely spread through lymphatic channels to abdominal lymph nodes and further to the liver and retroperitoneal areas. However, aggressive mucinous adenocarcinomas can spread and be fatal (7).

Primary testicular mucinous cystadenoma must be distinguished from testicular teratoma and metastatic testicular mucinous cystadenoma. Testicular teratoma is the most common testicular tumor in children, typically appearing as a well-defined cystic or mixed lesion on ultrasound with minimal blood flow signals (8). Mature teratomas are cystic lesions with septa and fat echoes, while immature teratomas are mixed cystic-solid lesions often associated with cystic changes, hemorrhage, or calcification (9). Metastatic mucinous tumors from the gastrointestinal tract are more common and have a poor prognosis compared to primary testicular mucinous tumors. Clinical evaluation including CT, MRI, endoscopy, and tumor markers is essential to exclude metastatic mucinous tumors, necessitating differentiation from B-cell lymphomas (including trophoblastic tumors), colorectal cancer, gastric cancer, and pancreatic cancer (10). However, the clinical presentation of testicular mucinous cystadenoma is variable, potentially causing symptoms such as testicular mass and scrotal swelling (11). Diagnosis typically requires histopathological confirmation, with pathological examination remaining the gold standard.

Although there are currently no specific serum biomarkers available for diagnosing primary mucinous cystadenoma of the testis, general testicular tumor markers such as alpha-fetoprotein (AFP), human chorionic gonadotropin (hCG), lactate dehydrogenase (LDH), alpha-amylase, CA-125, carcinoembryonic antigen (CEA), placental alkaline phosphatase (PLAP), squamous cell carcinoma (SCC) antigen, neuron-specific enolase (NSE), and the beta subunit of hCG (HCG-β) are commonly used to exclude other types of testicular tumors (12) (13) (14). In cases of primary mucinous cystadenoma, these markers are typically within normal ranges (15) (16). Abnormal elevations may indicate consideration of other types of testicular tumors (17), as shown in **Table 1**.

**Table 1:** Serum markers assist in the diagnosis and monitoring of testicular tumors.

Serum biomarker	Normal range	Elevated levels may indicate	Expression in primary testicular mucinous cystadenoma
Alpha-fetoprotein (AFP)	< 10 ng/mL	Yolk sac tumor, teratoma	Typically normal
Human Chorionic Gonadotropin (hCG)	< 5 mIU/mL	Choriocarcinoma, some teratomas	Typically normal
Lactate Dehydrogenase (LDH)	140 - 280 U/L	Non-specific, elevated in various testicular malignancies	Typically normal
Alpha-Amylase	23 - 85 U/L	Seminoma	Typically normal
CA-125	< 35 U/mL	Ovarian cancer, some testicular tumors	Typically normal
Carcinoembryonic Antigen (CEA)	< 3 ng/mL (non-smokers)	Various cancers including testicular tumors	Typically normal
Placental Alkaline Phosphatase (PLAP)	< 70 U/L	Seminoma	Typically normal
Squamous Cell Carcinoma Antigen (SCC)	< 1.5 ng/mL	Squamous cell carcinoma, including testicular and other sites	Typically normal
Neuron-Specific Enolase (NSE)	< 12.5 ng/mL	Neuroendocrine tumors, including small cell lung cancer	Typically normal
HCG-β Subunit	< 2 mIU/mL	Trophoblastic tumors, some non-germ cell tumors	Typically normal

Elevations in serum carcinoembryonic antigen (CEA), cancer antigen 125 (CA-125), and carbohydrate antigen 19-9 (CA19-9) are inherently nonspecific and may be observed in gastrointestinal as well as other mucinous neoplasms (18). Although preoperative imaging did not reveal a definitive abdominal primary lesion, cross-sectional imaging alone is insufficient to exclude a gastrointestinal origin. Comprehensive evaluation, including upper and lower endoscopic examination, targeted repeat abdominal imaging, and, where feasible, histopathological confirmation of any suspicious lesions, is essential to enhance diagnostic accuracy. In this case, it is intriguing to observe elevated laboratory results for CEA, CA-125, and CA 19-9. This finding may prove beneficial for our future diagnostic efforts.

The limitations are substantial and should be acknowledged explicitly. The abdominal lesions were not biopsied and therefore were not pathologically confirmed. Immunohistochemical staining of the primary specimen was not performed. Complete gastrointestinal workup was not documented, so an extratesticular primary mucinous tumor cannot be excluded. In addition, the possibility of under-sampling of a borderline or malignant component within the orchiectomy specimen cannot be excluded. These limitations prevent a definitive conclusion that the patient had a biologically benign mucinous cystadenoma with proven abdominal metastases.

Due to the rarity of cases, standardized staging and treatment protocols have not been established. Most tumors are detected while still localized to the testis, with the primary treatment being surgical debulking, such as radical orchiectomy, followed by close clinical monitoring (12).

## Data Availability

The original contributions presented in the study are included in the article/supplementary material. Further inquiries can be directed to the corresponding author.
